# Gut microbiome-mediated mechanisms in aging-related diseases: are probiotics ready for prime time?

**DOI:** 10.3389/fphar.2023.1178596

**Published:** 2023-06-01

**Authors:** Jing Ren, Huimin Li, Guixing Zeng, Boxian Pang, Qiuhong Wang, Junping Wei

**Affiliations:** ^1^ Department of Endocrinology, Guang’anmen Hospital, China Academy of Chinese Medical Sciences, Beijing, China; ^2^ Graduate School of Beijing University of Chinese Medicine, Beijing, China

**Keywords:** probiotics, inflammation, gut microbiota, intestinal barrier, dysbiosis, elderly

## Abstract

Chronic low-grade inflammation affects health and is associated with aging and age-related diseases. Dysregulation of the gut flora is an important trigger for chronic low-grade inflammation. Changes in the composition of the gut flora and exposure to related metabolites have an effect on the inflammatory system of the host. This results in the development of crosstalk between the gut barrier and immune system, contributing to chronic low-grade inflammation and impairment of health. Probiotics can increase the diversity of gut microbiota, protect the gut barrier, and regulate gut immunity, thereby reducing inflammation. Therefore, the use of probiotics is a promising strategy for the beneficial immunomodulation and protection of the gut barrier through gut microbiota. These processes might positively influence inflammatory diseases, which are common in the elderly.

## 1 Introduction

According to the United Nations, the global population aged ≥60 years is estimated to double to nearly 2.1 billion by 2050. Hence, population aging has become a global public health concern with important socio-economic issues (Jayanama and Theou, 2020). Senescence is characterized by increased concentrations of many pro-inflammatory factors in the circulation. In addition, chronic low-grade inflammation has been identified as a key process involved in aging (López-Otín et al., 2013). Inflammation is a normal part of the immune response that defends against harmful bacteria and pathogens and plays an important role in the maintenance of tissues (van de Vyver, 2023). Chronic low-grade inflammation is influenced by changes in different tissues (muscle, adipose tissue), organs (brain, liver), systems (immune system), and ecosystems (gut flora) (Calder et al., 2017). It can indirectly trigger diseases in other organs (e.g., metabolic diseases, neuroinflammatory diseases, cardiovascular diseases, etc.) (Hutchinson et al., 2020). Chronic low-grade inflammation is one of the main contributing factors to various age-related diseases in the elderly (Warman et al., 2022).

Furthermore, it is closely related to the dysregulation of gut flora (Calder et al., 2017). Many immune cells and microbiota in the digestive tract interact with each other to maintain immune homeostasis. Intestinal microbiota plays a role in maintaining healthy levels of inflammation by integrating gastrointestinal, immune, and neurological information (Belkaid and Hand, 2014; Estrada and Contreras, 2019; Hutchinson et al., 2020). Data obtained from animal models have demonstrated that age-related microbial ecological disorders can lead to intestinal permeability, systemic inflammation, and premature death (DeJong et al., 2020). Altering the gut microbiota of older adults with wholesome bacteria exerts positive effects on the maintenance of optimal immune responses, which decline with age. Such effects include delaying the aging of T lymphocytes and increasing the number of immune cells that respond to acute antigen exposure (Moro-García et al., 2013; Hutchinson et al., 2020). This article summarizes the current knowledge on gut flora and chronic low-grade inflammation, describes the effect and possible mechanism of probiotics on chronic low-grade inflammation in old age, and focuses on their potential use to improve the health of the elderly with systemic inflammation.

## 2 Dysregulation of gut flora and chronic low-grade inflammation

As an important part of the human microbiome, gut flora performs necessary biological functions, such as stabilizing the immune system, regulating host metabolism, preventing pathogen invasion, and improving epithelial barrier function (Malard et al., 2021; Rastogi and Singh, 2022). Studies have found that dysregulation of gut flora plays an important role in numerous immune-mediated chronic low-grade inflammatory diseases, such as old age weakness (Xu et al., 2021), type 2 diabetes mellitus (T2DM) (Gurung et al., 2020), obesity (Gomes et al., 2018), allergy (Cukrowska et al., 2020), cognitive dysfunction (Bairamian et al., 2022), non-alcoholic fatty liver (Safari and Gérard, 2019), ulcerative colitis (Shen et al., 2018), Crohn’s disease (Agus et al., 2018), irritable bowel syndrome (Ford et al., 2020), colorectal cancer (Wong and Yu, 2019), and cardiovascular disease (Witkowski et al., 2020). The ecological dysregulation of gut flora stems from the changes in the composition of gut microorganisms and gut barrier, leading to chronic low-grade inflammation.

### 2.1 Changes in the composition of gut flora

The composition of gut microbiota is considered a key factor for healthy gut physiology (Warman et al., 2022). The intestinal cavity contains billions of bacteria. Phyla Firmicutes, Bacteroidetes, Actinobacteria, and Proteobacteria account for 99% of the intestinal flora, while the remaining 1% consists of flora with other functions and benefits (Tagliabue and Elli, 2013). Some of these gut bacteria possess anti- or pro-inflammatory properties. Microbial strains with anti-inflammatory properties increase the production of short-chain fatty acids (SCFAs). SCFAs (butyrate, acetate, propionate, etc.) regulate energy metabolism and act as immunomodulators to maintain the anti-inflammatory/pro-inflammatory balance (Salazar et al., 2020). Microbial flora associated with increased SCFA production includes *Clostridium*, *Akkermansia*, *Lactobacillus*, *Lachnospira*, *Faecalibacterium*, *Bifidobacterium*, *Roseburia*, *Ruminococcus*, and *Dorea* (Myhrstad et al., 2020; Malesza et al., 2021). Among them, *Akkermansia muciniphila*, which belongs to the warty microbial phyla, is a mucin degradation strain that exists in the intestinal mucus layer and improves intestinal barrier integrity by enhancing mucin production and complex interactions with other bacteria (Malesza et al., 2021). Moreover, it supports gut colonization by beneficial bacteria producing SCFAs (Bodogai et al., 2018). *Bifidobacterium* spp. promotes the production of anti-inflammatory cytokines, such as tumor necrosis factor (TNF) and interleukin-1 beta (IL-1β), induces the maturation of immune cells, promotes immunoglobulin A (IgA) secretion, and possesses anti-oxidant properties (Toward et al., 2012; Malesza et al., 2021). Microbial strains with pro-inflammatory properties, such as *Escherichia coli*, *Eggerthella lenta*, *Streptococcus gallolyticus*, and *Enterococcus* spp., can produce more endotoxins (Warman et al., 2022).

#### 2.1.1 Inappropriate use of early antibiotics

Early life (including the embryonal period and infancy) is a critical time for the colonization and formation of gut flora. Disturbance of the gut flora during this period can have long-term health effects and may increase the risk of metabolic diseases such as obesity and T2DM. Intestinal flora colonization occurs mainly at birth and several days after birth as the fetus is relatively aseptic in the mother’s uterus. During delivery and breastfeeding, intestinal colonization occurs, which gradually establishes the infant’s intestinal flora (Cox and Blaser, 2015). In the first few weeks after birth, the microbiota in the body is similar to the mother’s vagina and skin flora, including *Enterococcus*, *Streptococcus*, *Lactobacillaceae*, *Clostridae*, and *Bifidobacteriaceae*. In addition, the flora in breast milk is also a source of intestinal flora in infants. Various dietary components, such as polysaccharides that cannot be digested by enzymes, can increase *Enterococcus*, *Clostridium*, and *Ruminococcus* and decrease *Bifidobacterium* and *Enterococcus* concentrations in the intestinal flora. As a result of the decrease in *Bifidobacteria*, the pH in the intestine increases, and the concentration of *Bacillus*, *Clostridium*, *Lactobacillus*, *Streptococcus*, and fungi increases. At the age of 1 year, the intestinal flora begins to resemble that of adults, and at the age of 3 years, it is basically similar to that of adults. Colonization of the intestinal flora resists the colonization and reproduction of pathogenic microorganisms and is essential for energy metabolism, growth and development, and maturation of the immune system in infants. The main factors affecting the establishment of gut flora early in life include intrauterine microbial exposure, delivery pattern, feeding pattern, diet, geographic environment, behavioral habits of infants and young children, and antibiotic use (Cox and Blaser, 2015). Among them, the effect of antibiotics in the resistance to pathogen invasion has been extensively examined. Additionally, antibiotic-induced microbiota interferes with host metabolism. Long-term high use of antibiotics can lead to changes in the structure of the gut symbiotic flora, leading to the growth of potentially pathogenic microorganisms, causing intestinal flora disorders and infection, thus, affecting host metabolism, resulting in the occurrence of metabolic diseases in adults (Cox and Blaser, 2015). Studies have shown that early intestinal microbial deviation precedes the development of obesity and overweight in childhood and adulthood, suggesting that changes in the composition of intestinal microbes that affect energy metabolism might begin in early life (Kalliomäki et al., 2008; Scheepers et al., 2015; Wilkins and Reimer, 2021). Studies have found that a reduction in the intestinal *Bifidobacteria* at the age of 3 months is associated with being overweight at the age of 10 years (Luoto et al., 2011). Aversa Z et al. have found that early antibiotic exposure was associated with an increased risk of childhood-onset asthma, allergic rhinitis, atopic dermatitis, celiac disease, overweight, obesity, and attention deficit hyperactivity disorder (Aversa et al., 2021).

Antibiotics are important for treating infectious diseases, but their long-term high use can lead to changes in the structure of the intestinal symbiotic flora, causing the growth of potentially pathogenic microbes and, thus, intestinal flora disorders and the emergence of drug-resistant strains (Wilkins and Reimer, 2021). The effect of antibiotics on the microbiome depends on the duration, dose, and frequency of treatment and age of the person (Vandenplas et al., 2020; Wilkins and Reimer, 2021). The greatest disruption to microbiota development occurs mainly in early life (McDonnell et al., 2021). According to statistics, 78% of mothers in Denmark received antibiotic treatment before, during, and 4 years after pregnancy (Stokholm et al., 2014). The use of antenatal antibiotics has been shown not only to disrupt the spread of microbiota from mother to child but also to have an impact on the birth weight of newborns associated with an increased risk of future obesity and related metabolic sequelae (Vidal et al., 2013). Antibiotics are widely used in infancy and childhood in the United States (Hicks et al., 2013). On average, American children receive nearly 3 antibiotics at age 2 years (McCaig et al., 2002). During the first 2 years of life in children exposed to antibiotics, the composition of the intestinal flora significantly changes, and the risk of obesity increases (Bailey et al., 2014; Wilkins and Reimer, 2021). In addition, because antibiotics are widely used to promote livestock growth, American infants might potentially be exposed to antibacterial agents from other sources, such as food supply chains or drinking water (Andersson and Hughes, 2014; Cox and Blaser, 2015). The extent to which these non-medical exposures affect the development of the early microbiome, as well as their impact on the health of the elderly, is an important topic for future research.

The development of microbiota in early life influences long-term metabolic function. Interference of antibiotics with the normal development of the gut microbiota can alter intestinal flora, usually only temporarily, but the metabolic consequences might persist over the long term or affect the health of the elderly, especially when animals experience obesity-causing diets, such as a diet high in fat or sucrose (Wilkins and Reimer, 2021). The use of broad-spectrum antibiotics significantly alters the composition of the gut microbiota, reducing diversity by more than 25%. However, once treatment is complete, changes in the microbiota will resume (Panda et al., 2014; Wilkins and Reimer, 2021). A worrying long-term effect of antibiotic use is their potential to produce antibiotic-resistant genes in microorganisms (Jernberg et al., 2010). Antibiotic resistance genes can alter insulin sensitivity, increase inflammatory cytokines levels, and alter SCFA metabolism and bile acid production, all of which represent potential mechanisms for microbiome-induced metabolic diseases (Montassier et al., 2021). Early use of antibiotics reduces the abundance of *Bifidobacteria*, the primary intestinal microorganism of newborns, and increases the ratio of Firmicutes/Bacteroidetes (Wilkins and Reimer, 2021). Increased prenatal exposure to antibiotics in infants, 3 and 12 months postpartum, is associated with childhood obesity (Tun et al., 2018). By reusing antibiotics in infants, feces collection for 24 months has found a decrease in the microbial co-abundance group (CAG) represented by *Fusarium oxysporum*, which is negatively associated with childhood obesity (Chen et al., 2020). A lifelong survey of antibiotic use in Finnish children has found that macrolide antibiotics were associated with changes in the composition of the microbiota, a decrease in actinomycetes, and an increase in agrobacteria and amoeba and had a positive correlation with body mass index (BMI) (Korpela et al., 2016). The gate level recovers after a year of withdrawal, but microbial richness decreases over time. In addition, the researchers have found that increased BMI was most correlated with antibiotic use when participants were exposed to prenatal antibiotics for ≥3 treatments (Zhang et al., 2019). Exposure to antibiotics early in life might lead to persistent and significant changes in the gut microbiota and alterations in metabolic function. It can be hypothesized that inappropriate use of early antibiotics leads to persistent and significant changes in the gut flora or might increase the risk of metabolic disease in adults or older adults.

#### 2.1.2 Intestinal flora associated with aging

As aging occurs, an increased abundance of pro-inflammatory flora can inhibit the growth of beneficial flora, leading to chronic low-grade inflammation and increasing the risk of various aging-related diseases. Many studies in animals and humans have shown that the composition of the gut microbiota varies with host age, with certain microorganisms having detrimental effects on health (Table 1). Compared with young individuals, the gut microbiota of the elderly is less diverse (Mangiola et al., 2018; Xu et al., 2021). Studies have shown that the abundance of *Bifidobacteria* and some members of *Firmicutes* with anti-inflammatory properties, including *Clostridium* cluster IV (*Ruminococcus obeum* et rel., *Roseburia intestinalis* et rel., *Eubacterium ventriosumet* rel., *Eubacterium rectale* et rel., and *Eubacterium hallii* et rel.), and *Clostridium* cluster XIVa (*Papillibacter cinnamovorans* et rel. and *Faecalibacterium prausnitzii* et rel.), was decreased in the elderly and centenarians (Biagi et al., 2010). In Italian centenarians, the abundance of beneficial bacteria (Ruminococcaceae, Lachnospiraceae, and Bacteridaceae families) found in the gut flora was decreased with aging (Biagi et al., 2016). In addition, studies have reported that the gut microbiomes of healthy elderly and healthy young individuals from the same population are similar in terms of composition (Bian et al., 2017). Yuping Yang et al. have found that *Rhodococcus* spp. was significantly abundant during the middle-old age stage (Yang et al., 2021). This genus contributed greatly to L-tryptophan, catechol, and inositol degradation pathways, as well as ectoine and L-arginine biosynthesis pathways. Gut bacteria-encoded functions, such as amino acid metabolism, B vitamin metabolism, aromatic compound metabolism, and energy metabolism, varied in an age-dependent manner, and *Rhodococcus* spp. was the most associated functional bacteria in middle-old-aged rats (Yang et al., 2021). As shown in Table 1, health-related genera, such as *Ackermanella*, *Bifidobacterium*, and *Christensonaceae*, and microbial diversity are enriched in long-living populations (Biagi et al., 2016; Kong et al., 2016). Despite changes in microbes in long-lived populations, their intestinal microbial diversity and beneficial microbes are preserved to support healthy aging. Overall, the composition of gut microbes changes while microbial biodiversity decreases as potential pro-inflammatory microbes accumulate, and beneficial microbes decrease with age. Therefore, maintaining the dominant position of beneficial bacteria in the gut might prevent chronic low-grade inflammation and promote healthy aging.

**TABLE 1:** The gut microbiota changes with aging.

Study model	Gut microbiota variations induced by aging	Pathophysiological mechanisms	References
*Drosophila melanogaster*	Gammaproteobacteria↑	Intestinal barrier dysfunction	Clark et al. (2015)
Turquoise killifish	Microbial diversity↓	Intestinal barrier dysfunction	Smith et al. (2017)
	Over-representation of pathogenic Proteobacteria↑		
Mice	Rikenellaceae family↑	Affects the bioavailability of B vitamins, other metabolites, and DNA repair function	Langille et al. (2014)
Mice	Bacteroidetes, Tenericutes↓	Induces the expression of p16 and activation of nuclear factor-kappa B (NF-κB)	Kim et al. (2016)
	Firmicutes, Actinobacteria↑		
Mice	Firmicutes/Bacteroidetes↑	Leads to an alteration in the metabolism	Vemuri et al. (2018)
Mice	Firmicutes (3–14 M↑, 20 M↓)	Causes a shift in metabolomic profiles	Luo et al. (2020)
	Bacteroidetes (3–14 M↓, 20 M↑)		
	Proteobacteria↑		
Mice	Firmicutes/Bacteroidetes↑	May be involved in the production of some pro-inflammatory metabolites	Shenghua et al. (2020)
Rats	Firmicutes/Bacteroidetes↓	N/A	Flemer et al. (2017)
Rats	Rhodococcus spp.↑	Gut bacteria-encoded functions	Yang et al. (2021)
Humans	Bacteroidetes↑	N/A	Claesson et al. (2011)
Humans	Firmicutes, Bifidobacteria↓	Intestinal barrier dysfunction	Rondanelli et al. (2015)
	Enterobacteriaceae, Bacteroidetes↑		
Humans	Firmicutes ↓	N/A	Odamaki et al. (2016)
	Bacteroidetes, Proteobacteria ↑		
Humans	Core microbiota ↓ (Ruminococcaceae, Lachnospiraceae, Bacteroidaceae)	N/A	Biagi et al. (2016)
	Subdominant species↑		
Humans	Firmicutes/Bacteroidetes↓	Lowers the anti-inflammatory and anti-cancer efficacy	Yu et al. (2020)
	Bifidobacterium, Eubacterium↓		
Humans (≥	*Bifidobacteria, Firmicutes*↓	N/A	Biagi et al. (2010)
90 years old)			
Humans (≥	Microbial diversity↑	N/A	Kong et al. (2016)
90 years old)	Several potentially beneficial bacterial taxa ↑ (*Clostridium* cluster XIVa, Ruminococcaceae, Akkermansia, Christensenellaceae)		

M, month; ↑, increase; ↓, decrease; N/A, the pathophysiological mechanisms associated with changes in gut microbes were not mentioned.

This age-related gut microbial disorder affects host health and longevity (Du et al., 2021). In fruit flies and mice, age-related microbiota disorders could lead to gut barrier dysfunction, a pathophysiological sign of aging (Clark et al., 2015; Thevaranjan et al., 2017). In addition, the genetic composition and metabolites of microorganisms can have a positive impact on the life of the host (Langille et al., 2014; Han et al., 2017). Therefore, gut microbes might play a regulatory role in aging.

### 2.2 Change in intestinal barrier integrity

The intestinal barrier is one of the largest and most important internal barriers of the body. It can protect the body from harmful substances and microorganisms present in the intestinal cavity. The gut barrier consists of immune cells in the mucus layer, gut flora, intestinal epithelial cells, and intrinsic layers (Kocot et al., 2022). The mucus layer is composed of mucoglycoproteins secreted by cup cells in the intestinal epithelium to prevent contact between microorganisms in the intestinal cavity and intestinal epithelial cells (Knoop and Newberry, 2018). Intestinal epithelial cells provide a physical barrier to prevent material leakage from the intestinal cavity (Vancamelbeke and Vermeire, 2017). The paracellular gap between intestinal epithelial cells is sealed by tight junctions (TJs), adhesion junctions, and desmosomes (Vancamelbeke and Vermeire, 2017; Buckley and Turner, 2018; Kocot et al., 2022). Adhesion junctions and desmosomes adhere directly to intestinal epithelial cells, while TJs lie between the sides of intestinal epithelial cells. TJs are composed of transmembrane proteins, including claudins, occludins, and peripheral membrane proteins (zonula occludens [ZO] and regulatory protein) (Buckley and Turner, 2018; Kocot et al., 2022). Impaired inter-cell connectivity can lead to increased intestinal permeability, thereby increasing the transport of inflammatory mediators and contributing to chronic low-grade inflammation. Immune cells in the intrinsic layer can secrete immunoglobulins, such as IgA, that bind bacteria and their toxins to prevent their translocation into the body (Allaire et al., 2018). The gut microbiota protects the integrity of the gut barrier through various functions, such as combating pathogen colonization and stimulating the production of mucus, antimicrobial proteins, and regulatory T (Treg) cells (Allaire et al., 2018).

Intestinal barrier dysfunction caused by changes in the intestinal barrier integrity is the core mechanism of metabolic disease and related gastrointestinal manifestations. Man et al. (2015) have found a decrease in the transepithelial electric resistance (TEER) of the living ileal tissue in the aging population. Age-related changes in the gut flora are closely related to increased gut permeability, leading to gut barrier dysfunction. Age-related ecological disorders of the gut microbiome, thinning of the mucin layer, and increased endothelial clearance are the reasons for the increased permeability of the mucosal barrier, which allows the translocation of microbes, toxins, and antigens into the circulation (Wilson et al., 2018). Gut barrier dysfunction can lead to immune cell infiltration and chronic low-grade inflammation of the gut mucosa, activating the immune response (Singh et al., 2021; Wei et al., 2021). Some studies have shown that the gut microbiome is an important part of neuroimmune crosstalk and that some immune cells, such as muscularis macrophages (MMs), act as intermediaries between ENS and gut microbes. Hunger of microbiota, for example, in the case of reduced fiber consumption, may increase microbiota dependence on mucopolysaccharides, leading to the degradation of mucus layers and increased susceptibility to pathogens, which further results in immune activation (Desai et al., 2016). *Ruminococcus gnavus*, *Akkermansia muciniphila*, and *Ruminococcus torques* are mucosa-associated bacteria. Some studies have shown that their changes are related to changes in mucus and gut secretions (Paone and Cani, 2020). In addition, under susceptible conditions, the consumption of certain pathogens or certain foods can cause a tightly linked change through proteasome-mediated degradation triggered by inflammatory mediators, such as proteases, aspartons, and histamines, which can disrupt the gut barrier function (Bischoff et al., 2014). MMs are immune cells that promote bone morphogenetic protein 2 (BMP2) expression and dependence on the secretion of colony stimulant 1 receptor (CSF-1R), a cytokine receptor. BMP2 stimulates the expression of BMP receptors I and II (BMPRI and BMPRII). The development of neurons and smooth muscle cells depends on BMP receptors. Studies have shown that when mice were treated with antibiotics, the expression of CSF-1R and BMP2 and MM number decreased. Hence, the microbiome is an important part of neuroimmune crosstalk during inflammation, with immune cells acting as an important intermediary between the ENS and gut microbiome (Muller et al., 2014). Metabolic disorders are closely related to chronic low-grade inflammation. Elevated levels of pro-inflammatory cytokines (TNF- α, IL- 1β, and IL-6) have been found in the circulation of obese mice and humans, causing insulin resistance and T2DM (Olefsky and Glass, 2010). Recently, a study published in Science has found that in T2DM and obese mouse models, hyperglycemia led to intestinal barrier dysfunction through the recombination of glucose transporter 2-dependent intestinal epithelial cells, altered tight junctions, and adhesion protein integrity (Thaiss et al., 2018). As a result of the high blood sugar-mediated destruction of the epithelial barrier, systemic inflows of microbial products can enhance the distribution of gut microbiome products, leading to intestinal infections. In addition, a recent study has characterized the age-related changes that occur to septate junctions (SJ) between adjacent absorptive enterocytes (EC) in the fly intestine. The study has found that acute loss of the *Drosophila* tricellular junctions (TCJ) protein gliotactin (Gli) in ECs led to rapid activation of stress signaling in stem cells and an increase in intestinal stem cell (ISC) proliferation. Furthermore, a gradual disruption of the intestinal barrier was observed (Resnik-Docampo et al., 2018).

Following dysregulation of the intestinal flora, the abundance of pro-inflammatory bacteria increases, whereas that of anti-inflammatory bacteria decreases. These changes affect the integrity of the intestinal barrier and exert harmful effects on the body. Accumulating epidemiological, experimental, and clinical data indicate a strong correlation between changes in intestinal barrier integrity and chronic low-grade inflammation. A study has found that the aging-related microbiome promoted intestinal permeability and inflammation and increased the levels of pro-inflammatory cytokines, such as IL-6 and TNF (Ticinesi et al., 2019). Fransen et al. (2017) have transplanted the gut flora of elderly mice into the gut of young sterile mice. They have found that decreased levels of *Akkermansia* and increased levels of TM7 bacteria and amoeba phyla led to increased intestinal permeability and inflammation. In addition, some bacterial metabolites (e.g., phenylacetic acid and trimethylamine) can induce pro-inflammatory cytokines, destroy intestinal barrier integrity, and promote chronic low-grade inflammation (Tilg et al., 2020).

**FIGURE 1:**
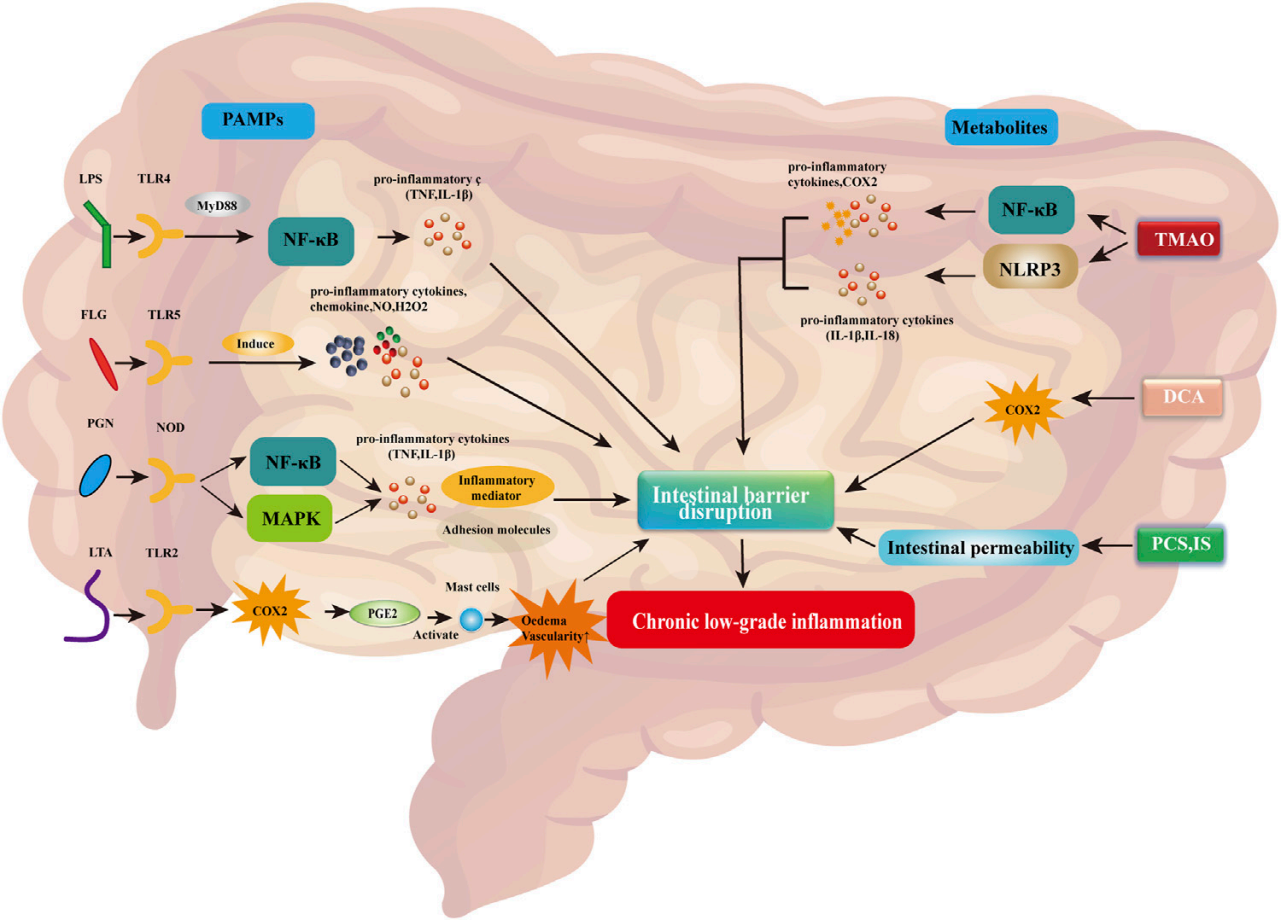
PAMPs and associated metabolites disrupt the intestinal barrier, thereby contributing to chronic low-grade inflammation. Abbreviations: COX2, cyclooxygenase 2; DCA, deoxycholic acid; FLG, flagellin; IL-1β/18, interleukin 1 beta/18; H_2_O_2_, hydrogen peroxide; IS, indoxyl sulfate; LPS, lipopolysaccharide; LTA, lipoteichoic acid; MAPK, mitogen-activated protein kinase; MyD88, myeloid differentiation factor 88; NF-κB, nuclear factor kappa B; NLRP3, NLR family pyrin domain-containing 3; NO, nitric oxide; NOD, nucleotide-binding oligomerization domain; PAMPs, pathogen-associated molecular patterns; pCS, p-cresol sulfate; PGE2, prostaglandin E2; PGN, peptidoglycan; TLR2/4/5, toll-like receptor 2/4/5; TMAO, trimethylamine N-oxide; TNF, tumor necrosis factor.

Pathogen-associated molecular patterns (PAMPs) are the main microbial factors contributing to chronic low-grade inflammation due to changes in intestinal barrier integrity caused by the dysregulation of gut flora. PAMPs are small molecules with conserved patterns, which are present in various microorganisms, including components that construct cell walls (bacterial peptidoglycan, lipopolysaccharide [LPS], lipoteichoic acid, and flagellate protein) and other factors common for microorganisms (viral RNA or DNA) (Dammermann et al., 2013). PAMPs are recognized by the pattern recognition receptor, which is present on the surface and in the cytosol of immune cells and intestinal epithelial cells (Schroder and Tschopp, 2010). Receptor families belonging to pattern recognition receptors include toll-like receptor (TLR), nucleotide-binding domain and leucine-rich repeat receptor (NLR), C-type lectin receptor (CLR), retinoic acid-inducing gene-I-like receptor (RLR), and absent in melanoma 2 (AIM2)-like receptor (ALR) (Potrykus et al., 2021). The binding of PAMPs to their respective specific receptors activates the innate immune system, thereby causing inflammation, enhancing damage to the intestinal barrier, and exacerbating chronic low-grade inflammation (Rajaee et al., 2018; Potrykus et al., 2021). A simplified representation of this process is shown in Figure 1.

LPS is a component of the extracellular wall of Gram-negative bacteria and can be identified by TLR4. Activation of this receptor leads to the release of pro-inflammatory mediators, including myeloid differentiation factor 88 (MyD88), which stimulates the production of pro-inflammatory cytokines (e.g., TNF and IL-1β) by the nuclear factor kappa-light chain-activated B cells (NF-κB) pathway (Gnauck et al., 2016; Potrykus et al., 2021). Flagelloprotein can activate TLR5. TLR5 is mainly expressed on the basolateral side of intestinal epithelial cells and detects the translocation of bacteria through the endothelial barrier (Yang and Yan, 2017). Activation of TLR5 by flagelloprotein leads to the synthesis of chemokines, nitric oxide (NO), hydrogen peroxide (H_2_O_2_), and pro-inflammatory cytokines (Hajam et al., 2017). Excessive activation of TLR5 leads to impaired intestinal barrier integrity and causes chronic low-grade inflammation (Yang and Yan, 2017). Peptidoglycan (PGN) is an important cellular structure that protects bacteria from environmental factors. It is recognized by PGN recognition protein, which is secreted by immune and epithelial cells and can dissolve bacterial cell walls. In addition, the identification of PGNs by nucleotide-binding oligomerization domain containing 1 (NOD1) and NOD2 receptors leads to the activation of NF-κB and mitogen-activated protein kinase (MAPK) pathways (Caruso et al., 2014). Both pathways contribute to the transcription of pro-inflammatory genes, leading to the synthesis of cytokines, adhesion molecules, and inflammatory mediators. Lipoteichoic acid is a component of the cell wall of Gram-positive bacteria. It is recognized by TLR2, thereby activating the respective signaling pathway. This process induces cyclooxygenase (COX) expression, which promotes the synthesis of prostaglandin E2 (PGE2) (Tominari et al., 2021). PGE2 causes edema and vascular permeability by activating mast cells, thereby promoting inflammation (Tominari et al., 2021).

Abnormal metabolites caused by gut flora dysregulation can also contribute to inflammation, enhance damage to the gut barrier, and exacerbate chronic low-grade inflammation. Trimethylamine N-oxide is a bacterial metabolite. Elevated trimethylamine N-oxide levels can induce the activation of the NLR family bilin-containing domain 3 (NLRP3). NLRP3 belongs to the inflammasome, which is the intracellular protein complex responsible for initiating inflammatory processes. Activation of NLRP3 activates caspase 1 (CASP1) to regulate the maturation and secretion of pro-inflammatory cytokines IL1B and IL18 (Potrykus et al., 2021). In addition, trimethylamine N-oxide activates NF-κB, thereby promoting the synthesis of pro-inflammatory proteins, such as COX2, selectin E (SELE), IL6, and intracellular adhesion molecule 1 (ICAM1) (Yang et al., 2019). Intestinal microorganisms cause bile acids in the intestines to detach and form secondary bile acids, such as deoxycholic acid. Elevated deoxycholic acid levels can lead to the overproduction of COX2, which is closely related to impaired intestinal epithelial integrity and intestinal inflammation (Zhao et al., 2016). In addition, high levels of bile acids contribute to the formation of reactive oxygen species and nitrogen substances. Moreover, toxic bacterial metabolites, such as p-cresol sulfate and indoxyl sulfate, can lead to increased intestinal permeability and exacerbate chronic low-grade inflammation (Li and Tang, 2018).

### 2.3 Chronic low-grade inflammation in aging

**FIGURE 2:**
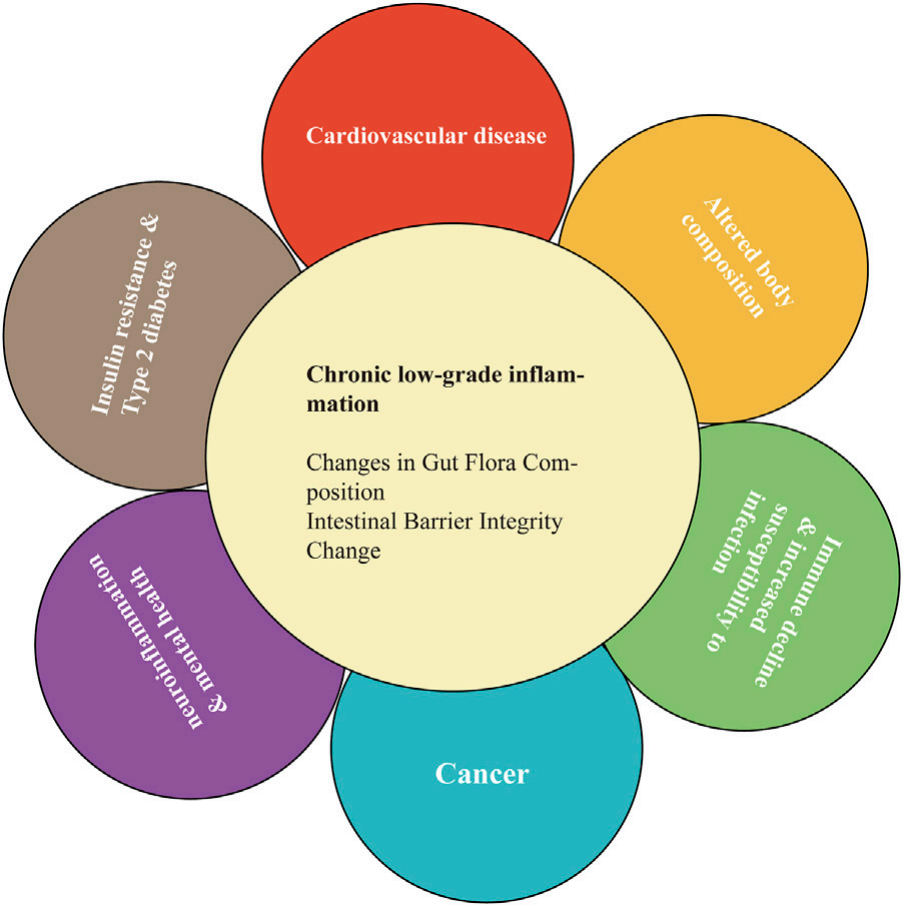
Chronic low-grade inflammation can promote age-related disease.

Chronic low-grade inflammation can promote the onset of age-related diseases, such as T2DM, obesity, metabolic syndrome, neurodegeneration, cardiovascular disease, and decreased immunity (Figure 2) (Calder et al., 2017). There is a significant relationship between mild inflammatory status and major diseases of the elderly (e.g., cardiovascular disease and T2DM), as well as disability and mortality (Calder et al., 2017; Mengozzi et al., 2021). Age-related chronic low-level inflammation can alter the relationship between the gut-related immune system and gut microbiota, leading to changes in the latter (Mello et al., 2016). A study has investigated the relationship between the composition of the gut microbiota and the inflammatory state of centenarians. Increased levels of IL-6 and IL-8 in peripheral blood were associated with the enrichment of amoeba phyla and a decrease in some butyric acid-producing bacteria (Biagi et al., 2010). Therefore, age-related modifications in the gut microbiota might contribute to chronic low-level inflammation on one side or be affected by systemic inflammation on the other side (Mello et al., 2016).

A previous study has reported that high levels of fibrinogen were associated with T2DM in older adults (Spazzafumo et al., 2013). A cohort study of older individuals in Italy has found that high circulating levels of inflammatory-related mediators, such as IL-6, interleukin-1 receptor antagonist (IL-1RA), and TNF receptor II (TNFR2), were associated with the occurrence of chronic diseases (i.e., hypertension, diabetes, ischemic heart disease, congestive heart failure, and cognitive impairment) (Fabbri et al., 2015). In addition, it was demonstrated that the baseline levels of IL-6 were markedly increased with aging. Furthermore, these alterations were significantly and independently correlated with a rapid increase in the incidence of multiple diseases over time (Fabbri et al., 2015). A meta-analysis of 31 cross-sectional studies has revealed significant associations of inflammatory markers, such as C-reactive protein and IL-6, elevated white blood cell counts, and fibrinogen levels with weakness as a health outcome (Soysal et al., 2017).

## 3 Probiotics, chronic low-grade inflammation, and aging-related diseases

The World Health Organization defines “probiotics” as living microorganisms that are beneficial to host health when present in sufficient quantities (Binda et al., 2020). Probiotic supplements directly influence the composition of gut flora through the introduction of healthy bacteria. Through the interaction with the gut microbiota, they influence the immune function of the whole body and mucosa and improve the gut barrier function (Jukic Peladic et al., 2021; Martel et al., 2022). In addition, probiotics can significantly reduce inflammatory biomarkers in middle-aged and elderly individuals (Warman et al., 2022). As mentioned above, chronic low-level inflammation is an important mechanism for age-related diseases. Studies in an elderly cohort (>65 years old) have revealed that administration of *Bifidobacterium* resulted in an increased abundance of this organism in stool samples, increased stool frequency, and reduced inflammatory status (Duncan and Flint, 2013). Lahtinen et al. have found that older adults who took *Bifidobacterium longum*, a longum fermented oat beverage for 6 months, had increased abundance of *B. catenulatum*, *B. bifidum*, and *B. breve* compared to the control group (Lahtinen et al., 2009). Bartosch et al.’s study has found that *Bifidobacterium bifidum* and *B. lactis*, given to the elderly population, increased Bifidobacteria abundance in the gut (Turchet et al., 2003). A recent meta-analysis has shown that probiotic interventions reduced IL-6 and C-reactive protein levels in middle-aged and older adults with chronic low inflammation (Custodero et al., 2018). Pan et al. (Pan et al., 2018) have observed that *Lactobacillus murinus* CR147 downregulated IL-8 production in TNF-stimulated Caco-2 cells and significantly increased the lifespan and the brood size of the *C. elegans*, indicating that the proliferation of *Lactobacillus murinus* in calorie-restricted mice causatively contributed to the attenuation of aging-associated inflammation. There is growing evidence in the literature describing the potential to regulate chronic mild inflammation through the use of probiotic supplements. Furthermore, the potential effects of these nutritional interventions on intestinal barriers and age-related diseases (e.g., metabolic diseases, cardiovascular diseases, neuroinflammation, and decreased immune function) have been emphasized.

Metabolic diseases and cardiovascular diseases frequently occur in the elderly (Khosla et al., 2020). Aging and obesity are major risk factors for chronic inflammatory diseases such as diabetes and atherosclerosis, while T2DM and its complications can seriously affect the quality of life of older persons. T2DM and cardiovascular disease are common metabolic diseases characterized by chronic low-grade inflammation. Recently, numerous studies have reported the beneficial effects of probiotics in alleviating obesity and T2DM. Probiotics can improve obesity by reducing lipid accumulation, oxidative damage, inflammation, and intestinal disorders. *Lactobacillus curvatus* HY7601 and *Lactobacillus plantarum* (*L. plantarum*) KY1032 have reduced overweight and fat accumulation in the high-fat diet mouse group while reducing the levels of inflammatory biomarkers (Park et al., 2013). *Lactobacillus*, particularly *Lactobacillus casei* (IMVB-7280 and IBS041), *Lactobacillus paracasei* (HII0 and CNCM I-4034), and *Lactobacillus rhamnosus* (*L. rhamnosus*; CGMCC1.3 and LA68), plays a positive role in reducing weight gain, cholesterol levels, obesity, and inflammation (Wiciński et al., 2020). In addition, probiotics might influence fat metabolism and the levels of alanine transaminase in obese rats (Karimi et al., 2015). Diabetes is a chronic metabolic disease characterized by a sustained increase in serum glucose. There is a significant correlation between inflammatory status and metabolic disorder in patients with diabetes (Rastogi and Singh, 2022). The consumption of probiotics has an effect on the composition of the microbiota of the intestinal tract. In turn, this reduces the intestinal epithelium and inhibits the immune response by blocking the TLR4 signaling pathway, ultimately increasing insulin sensitivity (Li et al., 2021; Rastogi and Singh, 2022). *L. plantarum* Y44 downregulates the expression of pro-inflammatory cytokine genes in the liver, intestine, and muscle tissue by activating the regulatory anti-inflammatory cytokine IL-10 (Liu et al., 2020). In streptozotocin-induced diabetic rats, the administration of *Lactobacillus fermentum* MCC 2759 was associated with decreased levels of glucose (detected through spectroscopy), pro-inflammatory cytokine IL-10, insulin sensitivity (glucagon-like peptide 1 [GLP-1], glucose transporter type 4 [GLUT-4], and adiponectin), and intestinal barrier integrity (ZO-1) and enhanced expression of TLR4 receptors (Qu et al., 2018). Although research on the role of probiotics in metabolic diseases has been mainly conducted in animal models or young people, clinical trials are still lacking, especially in older adults. However, considering obesity and T2DM as age-related diseases, probiotics should be beneficial for older patients with obesity and T2DM.

Cardiovascular disease is a major cause of death worldwide (Youssef et al., 2021). Aging is an important risk factor for cardiovascular disease. Systemic low-grade inflammation, altered gut microbiota composition, and increased gut permeability are important features of aging and are closely related to the occurrence of cardiovascular disease (Gargari et al., 2022). Probiotics enhance the basic functions of cardiovascular and metabolism-related organs by protecting the gut barrier, regulating chronic low-grade inflammation, and maintaining intestinal homeostasis. By regulating the gut microbiome in older mice (26–27 months old), consistent with that observed in younger mice (5–6 months old), arterial function was preserved, while vascular oxidative stress and inflammation weakened (Brunt et al., 2019). A study has shown that age-related ecological disorders of microorganisms contribute to vascular aging. In addition, aging is associated with a larger abundance of the genus Desulfovibrio, leading to increased plasma trimethylamine N-oxide (TMAO) levels and risk of atherosclerosis (Brunt et al., 2019). Giorgio Gargari et al. (Gargari et al., 2022) have found that polyphenols-rich diets reduce intestinal permeability, inflammation, and lipid abnormalities in older populations, thereby reducing cardiovascular risk. A study has found that *Akkermansia muciniphila* can effectively inhibit atherosclerotic damage in apolipoprotein E (ApoE)^−/−^ mice by increasing the expression of TJ protein, improving restoration of the gut barrier, and reducing endotoxin-induced inflammation (Li et al., 2016). A placebo-controlled trial has shown that high doses of *L. plantarum* (DSM9843) effectively reduced atherosclerotic plaques by altering gut flora and increasing SCFAs (Karlsson et al., 2010). Furthermore, hypertension is an important risk factor for cardiovascular disease. Specific probiotic strains, such as *Lactobacillus fermentum*, *Lactobacillus gasseri*, and *Lactobacillus coryniformis*, have shown their ability to prevent hypertension and endothelial dysfunction in rat models, as well as to promote innate immunity. In addition, they can inhibit inflammation by restoring the balance between auxiliary T helper 17 (Th17) and Treg cells (Toral et al., 2018). Moreover, high-density lipoproteins reduce the risk of cardiovascular disease by limiting the accumulation of low-density lipoproteins in the blood vessel walls. *L. plantarum* can help reduce the levels of total cholesterol and triglycerides and improve hypercholesterolemia by increasing the concentration of high-density lipoproteins and lowering that of low-density lipoproteins (Lee et al., 2018). In general, probiotics have a significant impact on the prevention and treatment of cardiovascular diseases.

The effect of probiotics on neuroinflammation mainly depends on the exchange of nerve, hormone, and immune signals between the gastrointestinal tract and central nervous system, i.e., the “intestine-brain axis.” Among them, Alzheimer’s disease (AD) and Parkinson’s disease (PD) are the most common, as they are observed in one-tenth of people aged >65 years (Hansson, 2021). Recent advances in microbiome research help understand two-way gut-microbiome-brain communication, revealing possible links between intestinal ecological disorders and neuroinflammatory diseases (Roy Sarkar and Banerjee, 2019). There is evidence indicating that probiotics and their beneficial metabolites play a significant role in reducing neuroinflammatory and neurodegenerative diseases in experimental models or clinical settings, including multiple sclerosis, PD, and AD. AD, the most common neurodegenerative disease, is the leading cause of dementia in the elderly, accounting for about 60%–80% of total cases. The intestinal microbiota of patients with AD significantly differs from that of healthy individuals. It is characterized by a reduced abundance of Firmicutes and Bifidobacterium and an increased abundance of Bacteroidetes (Brunt et al., 2019). Similar microbiological changes have been reported in healthy older adults and might be a potential contributor to age-related cognitive impairment tendencies (Kong et al., 2019). *Lactobacillus mucosae* NK41, *Lactobacillus reuteri* NK33, *Bifidobacterium longum* NK46, and *Bifidobacterium adolescentis* NK98 can block the NF-κB pathway and reduce the levels of LPS, corticosterone, IL-6, and TNF in serum, thus, decreasing stress-induced anxiety/depression in mice (Jang et al., 2019). Additionally, *Lactobacillus acidophilus*, *Bifidobacterium longum*, and *Bifidobacterium bifidum* enhance spatial learning and memory in rats (Rezaei Asl et al., 2019). PD is one of the most common neuroinflammatory diseases, with aging considered the most important risk factor because the median age for PD onset is 60 years (Simon et al., 2020). Most patients with PD suffer from gastrointestinal diseases such as constipation, nausea, and vomiting, as well as increased intestinal permeability. Studies have found that giving multi-species supplements containing strains of *Lactobacillus*, *Bifidobacterium*, and *Streptococcus* to older adults with PD led to better bowel habits (Barichella et al., 2016) and reduced abdominal pain and bloating (Georgescu et al., 2016). Moreover, multiple sclerosis is another age-related neuroinflammatory disorder. Studies have reported that oral *Lactobacillus casei*, *Lactobacillus acidophilus*, and *Lactobacillus reuteni* delay the progression of multiple sclerosis by increasing the number of forkhead box P3 (Foxp3)^+^ and IL-10^+^ Treg cells and reducing pro-inflammatory Th1/Th17 polarization in the peripheral immune system and inflammatory sites (Kwon et al., 2013).

Decreased immune function is associated with chronic low-grade inflammation. This decreased immune function includes the loss of B and T lymphocytes, Th2-type cell-mediated immune response polarization, and changes in myeloid cell recruitment and phagocytosis that lead to increased susceptibility to numerous age-related diseases (Calder et al., 2017). Decreased immune function and chronic low-grade inflammation are also significant features of aging and age-related diseases, such as respiratory tract infections and cancer. Probiotics exert immunomodulatory effects, improving the decline in immune function related to those diseases. The ability of probiotics to regulate the immune system has been studied with the aim of preventing and/or limiting the effects of immune aging (Sharma et al., 2014). Several strains of Bifidobacterium and *Lactobacillus* probiotics have had a positive effect in reducing inflammation and the duration of winter infections in the elderly population (Ouwehand et al., 2008). Both *Lactobacillus rhamnosus* HN001 and *Bifidobacterium lactis* HN019 have increased natural killer cells and phagocytic activity in healthy elderly subjects (Mello et al., 2016). They also regulate lung immunity and promote respiratory health by regulating the intestinal-lung axis in both directions. A previous study has reported that *L. rhamnosus* CRL1505 increased the levels of TNF, interferon beta (IFN-β), IFN-α, and IFN-cytokine in the lungs, thereby significantly reducing respiratory inflammation (Kolling et al., 2018). In a model of allergic asthma, *L. rhamnosus* GR-1 has protected the gut barrier by regulating Th2-mediated immune responses and altering the composition of the gut microbiome. This probiotic passes through the gut-lung axis, thus, significantly reducing the severity of airway inflammation and hyperactivity (Spacova et al., 2020). In addition, certain strains of *Lactobacillus* secrete metabolites, particularly SCFAs (e.g., acetate, propionate, and butyrate), which can regulate the immune response of the host lung (Koh et al., 2016).

Cancer is the second leading cause of death worldwide after cardiovascular disease. Most patients with sporadic cancers are over 50 years old, and 75% of rectal cancer patients and 80% of colon cancer patients are over 60 years old (Haraldsdottir et al., 2014). Increased local inflammatory response, leading to the loss of intestinal barrier integrity and systemic inflammation, is one of the pathological mechanisms responsible for gastrointestinal cancer (Irrazábal et al., 2014). Intestinal microbial ecological disorders and the subsequent development of pathogenic flora may affect host metabolism or host intestinal and immune system function, leading to tumor growth (Vivarelli et al., 2019). However, some probiotic strains can directly regulate immune responses. Injection or oral administration of *Bifidobacteria* can effectively regulate the anti-cancer immune response in mice and inhibit cancer growth (Abdolalipour et al., 2022). Studies have shown that specific probiotic strains inhibit variants of common pathogens in the gut, such as *Escherichia coli*, *Salmonella intestinalis*, and *Clostridium pneumoniae* (Górska et al., 2019; Sehrawat et al., 2021). These pathogens secrete enzymes that convert pre-carcinogens into carcinogens, such as β-glucosylase, azo-reductase, and nitro-reductase (Pellegrini et al., 2020).

These studies have suggested that altering the gut microbiota of older populations by ingesting probiotics might be an effective strategy against aging and age-related diseases. At the same time, probiotics might be suitable and affordable for most older people. However, their health effects are complex, depending on the individual population and the duration of treatment. For reasons of effectiveness and safety, the development of probiotics beneficial to human health must consider possible high individual differences.

## 4 Mechanism of action of probiotics

**FIGURE 3:**
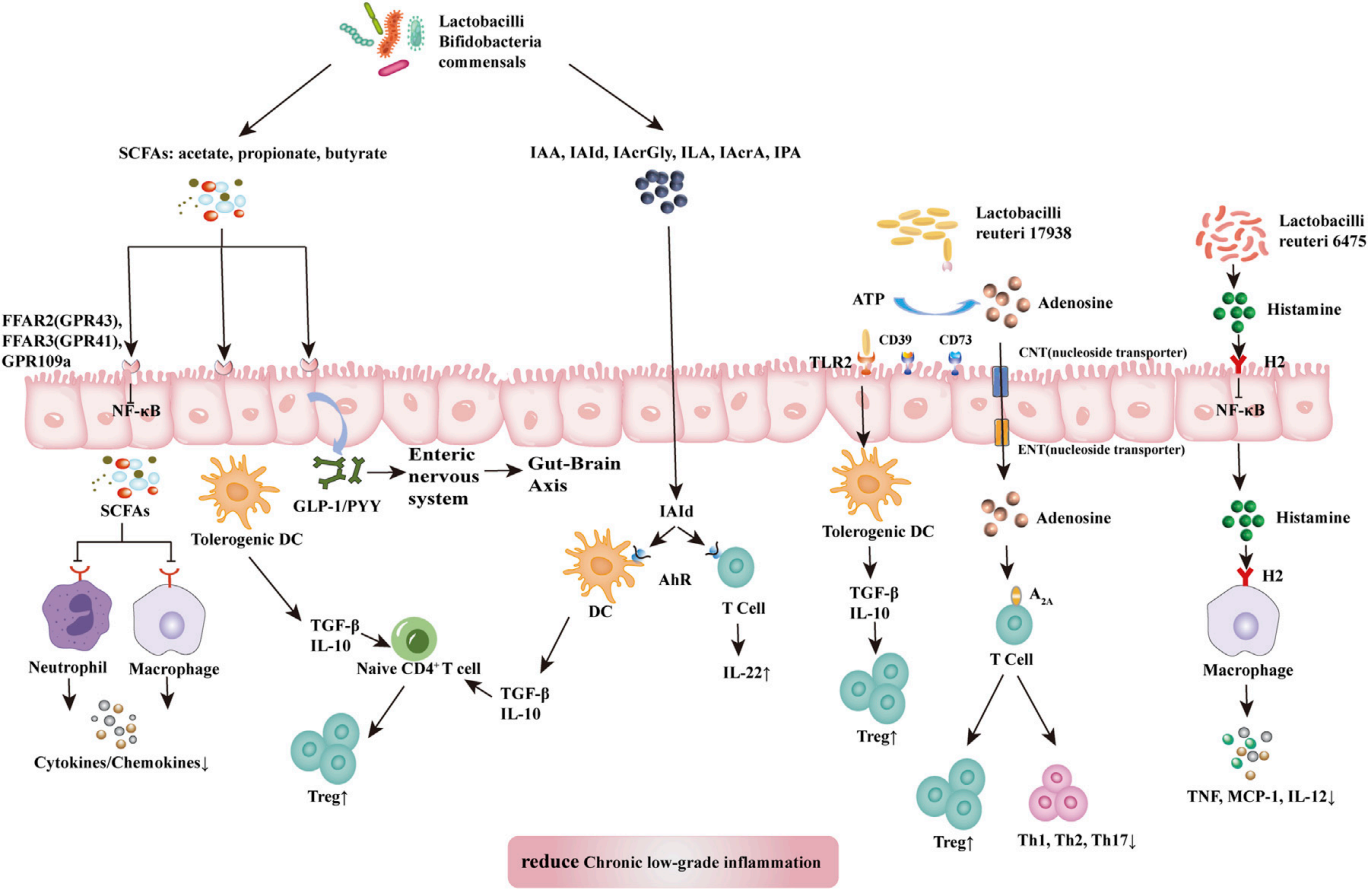
Mechanism of probiotic inhibition of chronic low-grade inflammation. SCFAs (acetate, propionate, and butyrate) produced by lactobacilli, bifidobacilli, and commensals bind and activate receptors (FFAR2, FFAR3, or GPR109a) on intestinal epithelial cells to inhibit the nuclear factor kappa-light-chain-enhancer of activated B cells (NF-κB) pathway to prevent inflammation. They also inhibit histone deacetylases from promoting the accumulation of Tregs and might release GLP1/PYY to act on the enteric and central nervous systems to influence energy homeostasis and gut motility. SCFAs also induce tolerogenic DCs, which educate naïve CD^4+^ T cells to differentiate into Tregs. These actions inhibit cytokine production by neutrophils and macrophages via interaction with receptors. Dietary tryptophan and probiotic-produced indole derivatives interact with AhR expressed on immune cells to produce anti-inflammatory effects. *L. reuteri* 17938 promotes adenosine generation, most likely by an ectonuclease present on the probiotic itself and intestinal epithelial cells. Adenosine and its derivative inosine interact with adenosine receptor-2A located on T cells to promote Treg functions and inhibit inflammatory Th1 and Th17 subsets. Histamine produced by *L. reuteri* 6,475 interacts with H2 presented on intestinal epithelial cells and macrophages to reduce levels of proinflammatory cytokines (TNF, MCP-1, and IL-12). In summary, the critical metabolites produced by probiotics generate anti-inflammatory effects in disease. Abbreviations: SCFAs, short-chain fatty acid; A_2A_, adenosine-inosine receptor 2A; AhR, aryl hydrocarbon receptor; FFARs, free fatty acid receptors; GLP-1, glucagon like protein 1; GPRs, G-binding protein receptors; H2, histamine receptor 2; IAA, indole-3-acetic acid; IAcrA, indole acrylic acid; IAcrGly, indole acryloyl glycine; IAId, indole-3-aldehyde; ILA, indole lactic acid; IL-10/12/22, interleukin 10/12/22; IPA, indolyl propionic acid; MCP-1, monocyte chemoattractant protein-1; NF-κB, nuclear factor kappa B; PYY, peptide YY; SCFAs, short-chain fatty acids; Th1/2/17, T helper 1/2/17; TNF, tumor necrosis factor.

Probiotics suppress chronic low-grade inflammation. The health-promoting effects of probiotics are attributed to the maintenance of a balanced abundance of Firmicutes, Bacteroidetes, Actinobacteria, and Proteobacteria, protection of the gut barrier, and achievement of an optimal immune balance (Tsai et al., 2019). Studies have shown that some probiotics can achieve effective therapeutic results by shifting the composition of microbiota to a more balanced structure (Pandey et al., 2015; Liu et al., 2018). Probiotics influence all components of the gut barrier, including the gut microbiome, mucus barrier, epithelial cells, the intrinsic layer rich in lymphocytes and plasma cells, vascular and neural components of the intrinsic layer, and mesenteric lymph nodes linked to the systemic immune system (Liu et al., 2018). Probiotics can produce key metabolites with anti-inflammatory effects, such as SCFAs, L-tryptophan (Trp) metabolites, adenosine, and histamine, that can regulate local and systemic metabolites and inhibit chronic low-grade inflammation (Figure 3).

SCFAs, specifically acetate, propionate, and butyrate, are produced by commensal bacteria (e.g., *Eubacterium rectale*, *Facecalibacterium prausnitizii*, *Eubacterium hallii*, and *Ruminococcus bromii*) and numerous probiotics (e.g., *Lactobacilli* and *Bifidobacteria*) (Liu et al., 2018). SCFAs have anti-inflammatory effects acting through a variety of mechanisms. SCFAs reduce the production of cytokines by neutrophils (Vinolo et al., 2011) while reducing macrophageal NF-κB signaling (Park et al., 2007). These effects inhibit chronic low-grade inflammation. Free fatty acid receptors (FFARs) and G protein-coupled receptors (GPRs) are SCFA receptors present in the colon. Among them, FFAR3 (GPR41) and FFAR2 (GPR43) on colon cells have been associated with movement control (Dass et al., 2007). SCFAs can bind and activate FFAR3 (GPR41) and FFAR2 (GPR43) located in the intestinal epithelium to induce the release of GLP-1 and peptide YY (PYY) into the basolateral environment (Liu et al., 2018). Released GLP-1 and PYY activate the intestinal nervous system and transmit neural information through the intestine-brain axis to the central nervous system, thereby influencing metabolic energy consumption by the host (Kuwahara, 2014). Treg cells are a subgroup of T cells with significant immunosuppressive effects that express Foxp3^+^, CD25^+^, and CD4^+^ as phenotypes. In addition, SCFAs (particularly butyrate) can induce the differentiation of Treg cells to inhibit chronic low-grade inflammation (Kespohl et al., 2017). Nevertheless, further study is warranted to identify the molecular mechanisms underlying these effects.

Trp metabolism plays a vital role in regulating intestinal immunity and protecting intestinal barriers (Hubbard et al., 2015). Probiotics inhibit chronic low-grade inflammation by regulating the intestinal barrier through Trp metabolites. These metabolites include bacteria-derived Trp metabolites (e.g., indole, indolic acid, skatole, and tryptamine), as well as host-derived Trp metabolites (e.g., kynurenines, serotonin, and melatonin) (Liu et al., 2018) (Gao et al., 2018). Trp metabolites can bind to aromatic hydrocarbon receptor (AhR). AhR is a cytoplasmic ligand-activated transcription factor in dendritic and T cells, which is involved in maintaining gut immune tolerance and barrier function. Host and bacterial Trp metabolites stimulate AhR and AhR-dependent gene expression, thereby producing IL-6, IL-22, vascular endothelial growth factor A (VEGFA), prostaglandin-endoperoxide synthase 2 (PTGS2), mucin 2 (MUC2), and cytochrome P450 1A1 (CYP1A1) and regulating intestinal homeostasis (Gao et al., 2018). Among them, indole acid derivatives from probiotics and symbiotics (e.g., indole-3-acetic acid, indole-3-aldehyde, indole acryloyl glycine, indole lactic acid, indole acrylic acid, and indolyl propionic acid) are considered the main metabolites involved in this process. Indole 3-propionic acid significantly enhances the production of the anti-inflammatory cytokine IL-10 after LPS stimulation and reduces TNF production (Venkatesh et al., 2014). Indole-3-aldehyde can activate group 3 innate lymphoid cells through AhR to produce IL22, thus, suppressing inflammatory responses (Zelante et al., 2013).

Probiotics inhibit the differentiation of Th1 and Th2 cells and improve chronic low-grade inflammation by altering the microbiota adenosine-inosine receptor 2A (A_2A_) axis (He et al., 2017a; He et al., 2017b). Aging can reduce the number of Treg cells and is closely associated with chronic low-grade inflammation. Transforming growth factor-beta (TGFβ) is a key immunomodulator in the intestinal mucosa. It can induce gene transcription in Foxp3^+^ thymic Treg precursor cells and convert initial T cells into induced Treg cells while protecting the latter cells from apoptosis. *Lactobacillus gasseri* SBT2055 activates TLR2 signaling, induces TGFB expression in dendritic cells, increases IgA production, and inhibits inflammation (Sakai et al., 2014). In addition, *Lactobacillus reuteri* 17938 restores the serum levels of the purine metabolite inosine and downstream products xanthine and hypoxanthine, thereby altering the metabolic spectrum that is reduced in Treg cell deficiency (He et al., 2017b). A key mechanism of Treg cells is the control of inflammatory effector memory T cells, including the Th1, Th2, and Th17 subgroups. These pro-inflammatory T cell families are controlled by the interaction of adenosine (produced by Treg cells) with receptor A_2A_, which is highly expressed on T cells (He et al., 2017a).

The tolerogenic effects of lactobacilli are strain- and metabolite-dependent (Liu et al., 2018). Studies have reported that *L. rhamnosus* strain, which secretes low levels of histamine, can suppress the immune response. Notably, *Lactobacillus saerimneri* strain, which secretes high levels of histamine, mediates the inflammatory response (Thomas et al., 2012; Ganesh et al., 2018). In addition, *Lactobacillus reuteri* 6,475 produces histamine, which acts through histidine decarboxylase, relies on histamine H-2 receptors on intestinal cells to inhibit TNF synthesis *in vitro*, and exert its anti-inflammatory effects (Thomas et al., 2012; Gao et al., 2017).

## 5 The challenges of using probiotics

Probiotics (mostly within *Lactobacillus*, *Bifidobacterium*, *Lactococcus*, *Streptococcus*, and *Saccharomyces* genera) are globalized, popularized, and integrated into foods, cosmetics, and supplements (Hoffmann et al., 2014). However, studies have reported highly mixed results. Research and development of probiotics inherently necessitate multiple cycles of trial and error to identify health benefits. In the absence of prior mechanistic information, the results in a plethora of literature are sometimes conflicting, thus, complicating the formulation of evidence-based clinical guidelines for the use of probiotics (Veiga et al., 2020). That merits better evidence-based proof of the impacts that probiotics have on humans and their adverse effects (Suez et al., 2019).

Probiotics are likely to be used in inflammation-related diseases as a component of various treatment regimens. Probiotic efficacy is strain- and indication-specific. Individual-specific factors can also contribute to the heterogeneity of probiotic supplementation outcomes, including diet, age, and microbiota (Veiga et al., 2020). The extent to which probiotic microorganisms can persistently or transiently colonize the gut during supplementation varies between individuals, depending (among other potential factors) on their resident microbiome (Veiga et al., 2020). Recent studies have shown that colonization-resistant microbiomes are more resilient to probiotic interventions compared to colonization-permissive individuals (Veiga et al., 2020). A precise approach to probiotics might bridge this gap by addressing heterogeneity associated with probiotic strains, individuals, and their microbiomes. If the determinants and/or mechanism of the host reaction are determined, probiotics developed through a top-down approach might eventually become precision probiotics (Veiga et al., 2020). Precision probiotics will be used as a better candidate for precision medicine and nutrition, as individuals who might respond to them will be identified based on the phenotype or target chosen for probiotics. Precision probiotics can be used to stimulate the production of beneficial microbial metabolites, inhibit the production of harmful compounds, or restore the ecological balance of metabolic networks by introducing key species damaged after intestinal inflammation or exposure to antimicrobial agents (Suez et al., 2019; Veiga et al., 2020). Furthermore, the precise method of probiotics is still subject to several challenges. First, because of the heterogeneity of probiotic strains, the ability to provide clinicians and consumers with specific guidelines for strains and/or combinations that are effective in a given medical condition is limited by a lack of research (Veiga et al., 2020). Second, individual-specific methods are used to predict efficacy, which might require extensive individualized host data (including genetics, anthropometry, and immunoassay) and microbiome data (e.g., strain level composition, transcriptomics, and metabolomics) and identify biomarkers associated with predicting colonization resistance and/or health outcomes. In addition, since fecal samples do not accurately reflect the colonization and influence of the gut microbiota along the gastrointestinal tract during probiotic replenishment, there is a strong need to design non-invasive means for identifying compatible probiotic-individual matching, such as ingestible microengineered osmotic pills. Another important factor to consider is safety, as exogenous microorganisms might have unintended effects on the microbiome and might even harm the health of vulnerable subjects and lead to bacteremia or fungemia. Therefore, understanding the mechanisms by which exogenous probiotic microorganisms (whether traditional or new) interact with the host and microbiome is important for efficacy and safety (Veiga et al., 2020).

In general, traditional, widely used probiotics, such as *Bifidobacterium* spp. and *Lactobacillus spp*., have been selected either randomly or by gathering living experiences. While most of them show biological safety and ameliorative effectiveness, the general effects and functions on the amelioration of diseases are statistically marginal (Chang et al., 2019). On the other hand, traditional probiotics are not applied to specific diseases. Therefore, the identification and characterization of novel and disease-specific next-generation probiotics (NGP) are urgently needed (Chang et al., 2019). NGPs are individual bacterial strains that scientists have screened and isolated by using rapidly evolving gene sequencing tools and bioinformatics platforms to characterize the composition and function of the gut microbiota and microbiome, as well as their relationship to the amelioration of inflammation-related diseases (Suez et al., 2020). The characteristics of NGP include a comprehensive understanding of their target disease, as well as the genetic and physiological characteristics of bacteria, including growth dynamics and antibiotic sensitivity patterns. In addition, its potential molecular improvement mechanism remains to be clarified (Chang et al., 2019). The introduction of next-generation techniques has considerably improved our ability to address the colonization question even at strain resolution to differentiate between endogenous and exogenous bacteria (Suez et al., 2020). NGP can better clarify probiotic colonization, as well as direct or microbiome-mediated effects on human hosts, and improve understanding of their mechanisms of activity, efficacy, and long-term safety.

## 6 Dietary modulation and gut flora

There is plenty of evidence that many foods, nutrients, and non-nutritious foods can regulate chronic low-grade inflammation by adjusting the gut flora (Minihane et al., 2015). The role of dietary patterns, specific foods, and individual nutrients and non-nutrients in influencing chronic low-grade inflammation has been extensively reviewed (Calder et al., 2011). Healthy diet patterns described by Healthy Diet Index, Alternative Healthy Diet Index, vegetarian diet, and Mediterranean diet (MD) are all associated with lower circulatory concentrations of inflammatory markers, including C-reactive protein (CRP) and several cytokines (Calder et al., 2017). Higher intake of whole grains, vegetables and fruits, nuts, and fish in healthy diets is associated with lower inflammation and overall wellbeing due to the presence of phenolic compounds and fiber (Calder et al., 2011). It is also a key feature in the prevention of non-communicable diseases (NCDs) through its impact on the microbiota. Several types of polyphenols can promote the growth of healthy gut microbial flora (e.g., *Bifidobacterium*, *Lactobacillus*, *Akkermansia*, *Christensenellaceae*, and *Verrucomicrobia*), and potential anti-aging effects have been reported. The intake of lemon polyphenols limits the abundance of gut flora associated with aging (Beli et al., 2018). Dietary fiber leads to the production of key metabolites, such as SCFA (good for health), with the potential to alter gut microbiota and change metabolic regulation (Henning et al., 2018). Therefore, food components and dietary habits can modulate gut microbiota composition and intestinal barrier functions (Du et al., 2021).

The characteristics of the MD include (a) high consumption of vegetables, fruits, cereals (mainly whole grains), nuts, and legumes; (b) low consumption of saturated fats, sweets, and meat; (c) high intake of unsaturated fats (especially olive oil); (d) medium-high fish consumption; (e) drinking wine in moderation; (f) medium-low intake of dairy products (mainly yogurt and cheese) (Klement and Pazienza, 2019). A study has found higher levels of SCFA in subjects who adhered to MD patterns better (Garcia-Mantrana et al., 2018). In addition, the abundance of *lactobacillus* in MD-fed monkeys has increased tenfold compared to Western diet-fed monkeys, which was accompanied by an increase in bile acid metabolites and a decrease in reactive oxygen metabolites (Shively et al., 2018). Thus, MD patterns can regulate chronic low-grade inflammation by regulating local microbiota.

A low-carbohydrate diet is an additional dietary approach associated with weight loss and improved health markers. In overweight individuals, a low-carbohydrate, high-protein weight loss diet has had no effect on the proportion of different bacterial phyla but triggered significant decreases in *Collinsella aerofaciens* and *E. rectale* relatives (Klement and Pazienza, 2019). A diet rich in complex carbohydrates increases levels of beneficial *Bifidobacteria*, such as subspecies *Bifidobacterium aureus*, *Bifidobacterium short*, and *Bifidobacteria polyformis*; on the other hand, it reduces levels of opportunistic pathogens, such as *Mycobacterium avium* subspecies paratuberculosis and Enterobacteriaceae (Walker et al., 2011). Low-carbohydrate diet facilitates the growth of anti-inflammatory microorganisms, such as Lachnospiraceae, while reducing pro-inflammatory microbes, such as *Bacteroides acidifaciens*, *Escherichia coli*, *Ruminococcus gnavus*, and *Clostridium cocleatum*. Hence, the low-carbohydrate diet has a positive effect on the gut microbial community, modulates chronic low-grade inflammation, and improves age-related diseases.

Ketogenic diets (KDs) are a special type of low-carbohydrate diet that reduce carbohydrate content to such an extent (usually <50 g/day) that the corresponding low insulin levels and mildly elevated cortisol levels induce the production of ketone bodies in the liver (Klement and Pazienza, 2019). In the mouse model of autism spectrum disorder, KDs increased the level of bacterium *A. muciniphila* and significantly increased the Firmicutes/Bacteroidetes ratio (Newell et al., 2016). In infants with refractory epilepsy, KD significantly alters the composition of gut microbes, bringing them close to healthy controls: *Bacteroides* and *Prevotella* increased, while *Cronobacter* levels decreased by approximately 50% (Xie et al., 2017).

Evolutional medicine argues that inadequate adaptation to modern lifestyles can lead to non-communicable diseases, and the paleolithic diet concept has been then introduced (Klement and Pazienza, 2019). The paleolithic diet refers to the modern diet that mimics the diet of our ancestors during the Paleolithic, which spans the majority of human existence in chronological order (Klement and Pazienza, 2019). Paleolithic diet typically consists of the following modes: (a) high consumption of fruits, herbs, spices, and vegetables; (b) moderate-to-high consumption of lean meat, organs, fish, and eggs; (c) appropriate consumption of nuts and seeds; (d) exclusion of all processed foods, legumes, cereals, dairy products, and vegetable oils (except for olive and coconut oils) (Klement and Pazienza, 2019). Healthy Italians, after more than a year of the modern paleolithic diet, have had much higher microbiome diversity than Italians who adhered to MD (Barone et al., 2019). The association with high microbiome diversity and its presumed anti-inflammatory properties will make the paleolithic diet an important complementary treatment in future clinical studies.

## 7 Conclusion

Chronic low-grade inflammation is thought to be responsible for many declining functions associated with aging and age-related diseases. Therefore, preventing, mitigating, or reversing inflammatory processes is highly correlated with healthy aging and improved wellbeing. It is important to understand the triggers of chronic low-grade inflammation and identify strategies to prevent, slow, or even reverse its development. Dysregulation of the gut flora is an important trigger of chronic low-grade inflammation. Changes in the composition of the gut flora and exposure to related metabolites directly interact with the inflammatory system of the host and develop crosstalk between the gut barrier and the systemic immune system. These processes contribute to chronic low-grade inflammation, resulting in poor health and impaired wellbeing associated with aging. Probiotics can effectively assist in maintaining the balance in the composition of gut microbial flora, thereby protecting the gut barrier and regulating gut immunity. Increasing scientific evidence has shown that probiotics exert a positive effect on chronic low-grade inflammation and play a key role in healthy aging and improving age-related diseases. Probiotics might be an important therapeutic strategy for the prevention, delay, or even reversal of low-grade inflammation in old age. However, robust controlled clinical trials are warranted to further validate this hypothesis.
